# Cystoscopy to investigate the prevalence of prostatic utricle cyst in boys with proximal hypospadias and its implications in medium-term follow-up

**DOI:** 10.3389/fped.2025.1514695

**Published:** 2025-02-26

**Authors:** Yuenshan Sammi Wong, Yuk Him Tam

**Affiliations:** ^1^ Division of Paediatric Surgery & Paediatric Urology, Department of Surgery, Prince of Wales Hospital, The Chinese University of Hong Kong, Hong Kong, Hong Kong SAR, China; ^2^ Department of Paediatric Surgery, Hong Kong Children’s Hospital, Hong Kong, Hong Kong SAR, China

**Keywords:** proximal hypospadias, prostatic utricle, prostatic utricle cyst, cystoscopy, differences of sex development

## Abstract

**Purpose:**

Prostatic utricle cyst (PUC) is known to be associated with hypospadias. We aimed to investigate the prevalence of PUC in children with proximal hypospadias by cystoscopy, and risk of symptom development after hypospadias repair.

**Methods:**

We retrospectively reviewed the records of patients who underwent 2-stage repair for proximal hypospadias and had cystoscopy for PUC assessment over the period of January 2017–September 2022.

**Results:**

A total of 34 patients with penoscrotal, scrotal or perineal hypospadias were included for review. First-stage repair was performed at a median age of 12 months. The median ventral curvature was 70 degrees (range 45–90). 4 patients had differences of sex development including 45,X/46,XY mosaicism (*n* = 2) and 46,XY partial gonadal dysgenesis (*n* = 2). PUC was detected by cystoscopy in 25/34(73.5%) patients, with longitudinal dimensions 10–35 mm (media*n* = 15 mm). At a mean follow-up of 30 months after hypospadias repair, 3/25(12%) patients developed symptoms associated with PUC including recurrent epididymoorchitis (*n* = 1), post-void dribbling (*n* = 1) and pyuria (*n* = 1). 3/10 PUC ≥20 mm became symptomatic compared with none of PUC <20 mm (*p* = 0.024). The patient with recurrent epididymoorchitis eventually underwent definitive surgery of PUC excision by robot-assisted approach.

**Conclusions:**

PUC is highly prevalent in proximal hypospadias. The use of the smallest-sized cystoscope as a screening tool can increase the diagnostic yield. Integrating cystoscopy in hypospadias surgery for concurrent PUC assessment can be considered as an option for patients with proximal hypospadias. Although the vast majority of PUC remains asymptomatic, those ≥20 mm in longitudinal dimensions may be associated with an increased risk of subsequent symptom development.

## Introduction

The prostatic utricle has been traditionally described as a cul-de-sac about 6 mm long that runs superiorly and posteriorly between the two ejaculatory ducts (1). It communicates with the posterior urethra at the verumontanum, and has a mixed embryological origin with its cranial and caudal part deriving from the Mullerian duct and urogenital sinus respectively (1, 2). Cadaveric studies suggest that the prostatic utricle is normally present to some extent in male populations (1). However, anomaly of the prostatic utricle is uncommon and has only been reported in 1% of men at autopsy (3). Incomplete regression of the Mullerian duct and/or decreased androgen stimulation of the urogenital sinus have been postulated to cause an enlarged prostatic utricle (2). There is a lack of clear differentiation between normal variants of the prostatic utricle and prostatic utricle anomaly in the literature. The terms enlarged prostatic utricle (2–5), prostatic utricle cyst (PUC) (6–8) or just prostatic utricle (9, 10) have all been used interchangeably in the literature to address an anomalous prostatic utricle. The authors of this study used the term “prostatic utricle cyst (PUC)” to describe the anomaly of an enlarged prostatic utricle.

The association of PUC with hypospadias and differences of sex development (DSD) has been known for several decades (2). The prevalence increases with increasing severity of hypospadias (11). Although the majority of PUC are asymptomatic, some people develop symptoms after hypospadias surgery. Symptomatic PUC presents with urinary tract infection (UTI), urinary dribbling, urinary retention, recurrent epididymoorchitis, stone formation and rarely, malignancy (3, 5–9).

PUC has been reported in 9%–67% hypospadias patients (2, 9–13). The wide range in the reported prevalence is attributable to variations in study subject selection and the choice of diagnostic modalities to assess PUC across different studies. For several years we had performed routine cystoscopy for PUC screening among patients with severe proximal hypospadias requiring 2-stage repair. We aimed to investigate the prevalence of PUC in boys with proximal hypospadias and the risk of symptom development after hypospadias surgery.

## Materials & methods

### Study subjects

Patients with proximal hypospadias who underwent hypospadias repair in our center over the period of Jan 2017–Sept 2022 were identified. We included in this study patients who had undergone 2-stage repair of hypospadias and cystoscopy for PUC assessment during the study period. In Sept 2017 we introduced cystoscopic screening for PUC to be performed concurrently with either stage of the 2-stage hypospadias repair, and since then all proximal hypospadias patients undergoing 2-stage repair in our center had concurrent cystoscopy. During the study period, we identified 31 patients who had received cystoscopic screening for PUC at the time of the 2-stage hypospadias repair. There were 3 other patients who had completed the 2-stage hypospadias repair in 2017 before the introduction of PUC screening, and had cystoscopy subsequently during the study period. A total of 34 patients were therefore eligible for inclusion in the present study. A retrospective review of the medical records of the study subjects was undertaken. Data including age at the time of surgery, karyotype, DSD workup, operative findings, symptoms related to PUC, treatment for PUC and follow-up were recorded. The extent of DSD workup varied from patient to patient. Standard investigations usually included karyotyping, biochemical tests, and radiological investigations. Molecular genetic tests were performed on a case-by-case basis. Testing modalities included single-gene testing by Sanger sequencing in the early study period, and targeted DSD-related gene panels or whole exome sequencing which subsequently became available. Chromosomal microarray or MLPA testing was performed to detect copy number variants if clinically indicated. Only those patients with chromosomal DSD or a firm diagnosis of specific condition of 46,XY DSD would be reported in this study. The study was approved by the clinical research ethics committee of our institution.

### Proximal hypospadias and surgical techniques

The authors defined proximal hypospadias by the meatus location at proximal penile shaft to perineum after degloving of the penis. During the study period, the authors used either tubularized incised plate urethroplasty, or 2-stage preputial flap repair for the correction of proximal hypospadias. Patients who had ventral curvature greater than 20 degrees despite full penile degloving and excision of dartos tethering on the ventrum would undergo 2-stage repair with transection of urethral plate in the first stage. The transected urethral plate with its native urethra was mobilized proximally along the corporal surface to straighten the penis. Any residual ventral curvature, if noted on artificial erection test, would be further corrected by dorsal midline plication. Urethroplasty in the second stage was typically closed in two layers and covered by tunica vaginalis flap.

### Cystoscopy for PUC screening and follow-up management

**Figure 1:**
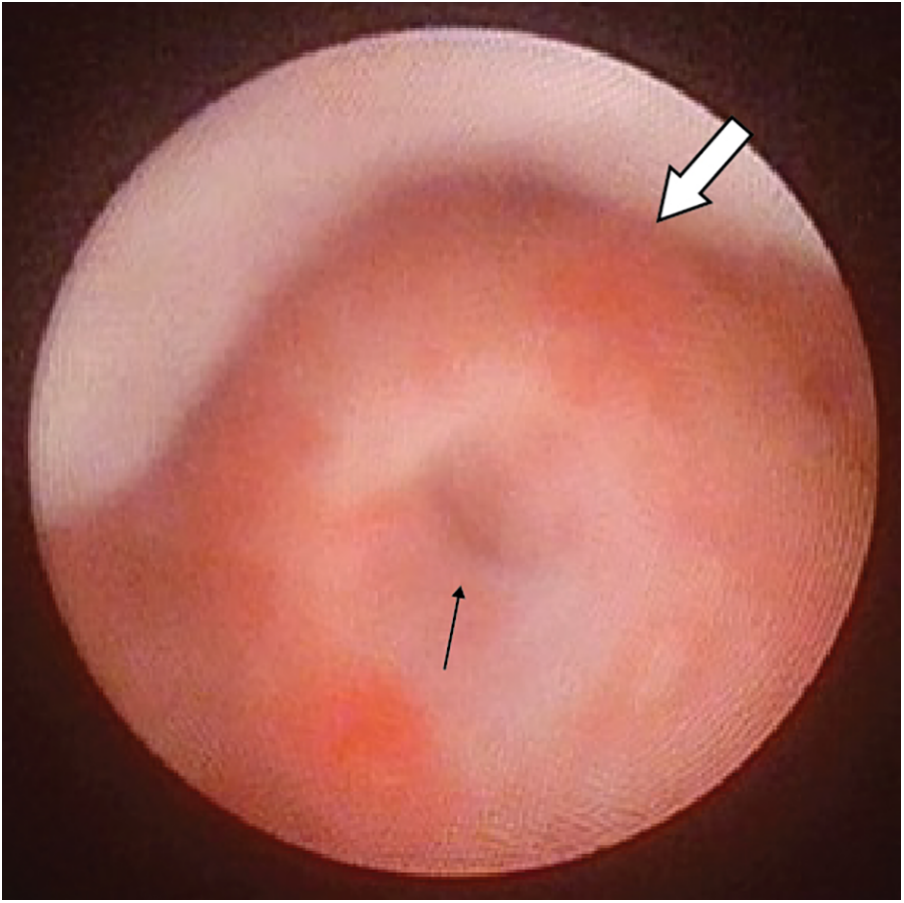
Cystoscopic view showing the opening of PUC(black arrow) at the roof of a normal verumontanum (white arrow).

**Figure 2:**
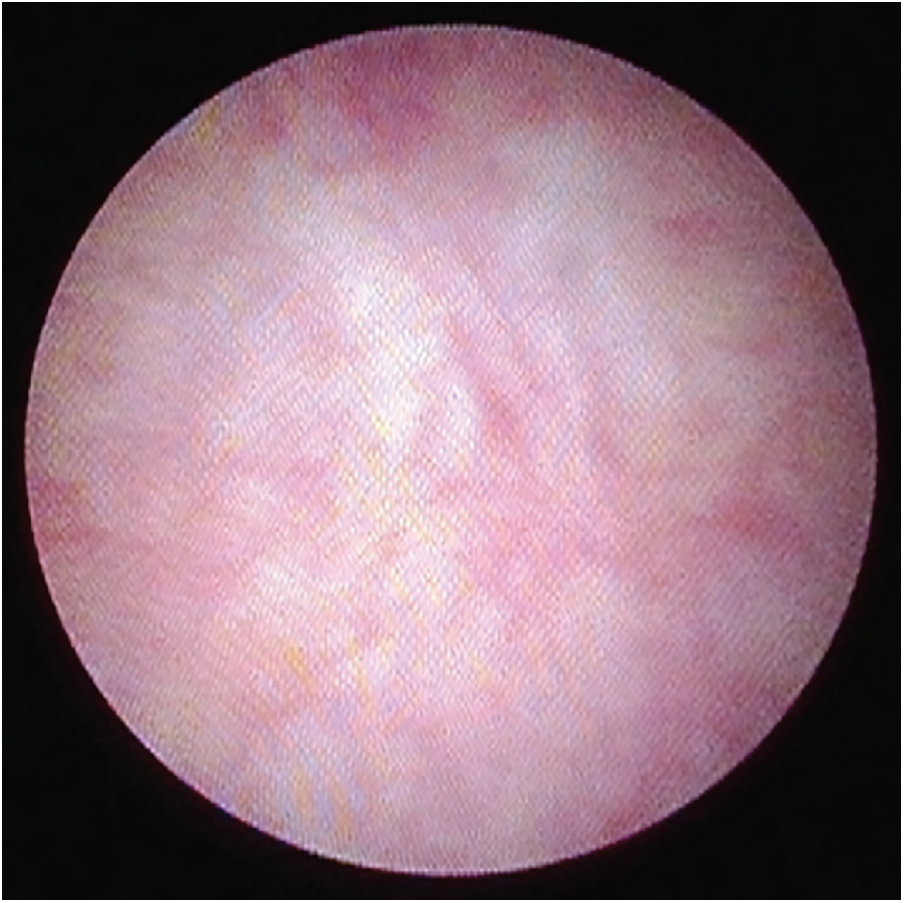
Cystoscopic view showing the inside of PUC cavity.

Cystoscopy for evaluation of PUC was performed using either a Fr 7.5 in early patients of the study, or Fr 4.5/6.5 rigid cystoscope which became available subsequently. To look for PUC, cystoscopic view focused at the roof of verumontanum. An opening noted at the roof of verumontanum would raise the suspicion of PUC, and positive finding was defined by having the scope to be able to pass through the opening into a cystic cavity (Figures 1, 2). The longitudinal dimension of the PUC was measured by the distance of withdrawing the cystoscope from the level when the instrument tip reached the dome of the cavity to the level when it was at the PUC opening. No additional procedure was performed for PUC detected by cystoscopic screening. Parents were informed of the PUC findings and its potential long-term complications. All proximal hypospadias patients with concomitant PUC were advised to have our long-term follow-up, and if indicated to receive transition care jointly with our adult urologists during their late adolescence. Repeat cystoscopy and/or radiological imaging for PUC reassessment would be offered if there were concerns about development of PUC-associated symptoms.

### Management for PUC-associated complications

Depending on the symptoms, conservative management which included antibiotics for bacterial complications remained to be the first-line approach. Radiological investigations such as ultrasound and micturating cystourethrogram were performed as per clinical indications. Cystoscopy was repeated on the grounds that it served both diagnostic and therapeutic purposes. It allowed evaluation for any interval progression in size of PUC, and any urethroplasty complications like strictures or diverticulum which predisposed to progressive urine stasis in PUC. Depending on the findings in repeat cystoscopy, therapeutic procedures such as dilation of urethral stricture or endoscopic widening of PUC opening and/or drainage were performed. Surgical excision of PUC was left as a last resort for symptomatic PUC which failed to respond to less invasive treatment modalities. All the cystoscopy, hypospadias repairs and surgical procedures for symptomatic PUC were performed by the two authors.

### Statistical analysis

Categorical data were expressed in percentage as frequency. Continuous data were expressed in mean with standard deviation (SD) or median with range. Patients with PUC detected were divided into two subgroups based on longitudinal dimension (<20 mm or ≥20 mm). Previous study has reported that majority of symptomatic PUC had a dimension of ≥20 mm measured by urethrogram (3). Categorical data were compared by chi-square test and continuous data were compared by Student *t* test. *P*-value < 0.05 was defined to be significant.

## Results

All 34 study subjects completed 2-stage preputial flap repairs for their proximal hypospadias during the study period. The first-stage repair was performed at a median age of 12 months (range 9 months–12 years) with a median interval of 9 months between the first and second stage. The mean ventral curvature was 71 degrees (SD = 16) before degloving. Meatus location was reported to be penoscrotal (*n* = 12), scrotal (*n* = 10) and perineal (*n* = 12). Of the whole group, 4 patients had DSD including 45,X/46,XY chromosomal DSD (*n* = 2) and 46,XY DSD (*n* = 2). The two 46,XY DSD patients had copy number loss at 9p24.3p24.1 and 9p24.3p23 respectively detected by microarray analysis. They both had the molecular diagnosis of *DMRT1* gene deletion which is a known etiology for 46,XY DSD due to gonadal dysgenesis (14). They were born with proximal hypospadias and micropenis. Testicular biopsies revealed seminiferous tubules composed of Sertoli cells only. Their phenotypes and histological features were compatible with partial gonadal dysgenesis.

PUC was detected by cystoscopy in 25 out of 34 (73.5%) patients. The longitudinal dimensions of PUC ranged from 10 to 35 mm (median = 15 mm). At a mean follow-up of 35 months (SD = 13) after staged hypospadias repair, 3 out of 25 (12%) patients developed symptoms which were possibly associated with PUC. The 25 patients with PUC were divided into two subgroups of PUC <20 mm (*n* = 15) and PUC ≥20 mm (*n* = 10). There was no difference in the mean ventral curvature (*p* = 0.47) or in the meatal locations (*p* = 0.11) between the two subgroups. Three out of 10 patients with PUC ≥20 mm developed symptoms compared to none in the subgroup of PUC <20 mm (*p* = 0.024). The 3 symptomatic patients had PUC dimensions of 20, 30 and 35 mm respectively. Two of the 10 patients with PUC ≥20 mm had abnormal verumontanum on cystoscopy revealing hypoplasia (*n* = 1) and absence (*n* = 1). Table 1 summarizes the characteristics of the study subjects.

**Table 1:** Summary of the characteristics of the study subjects.

Study parameters	Proximal hypospadias patients who had PUC screening by cystoscopy *N* = 34	Proximal hypospadias patients with positive finding of PUC on cystoscopy, *N* = 25 (25/34; 73.5% of the whole study group)	
		Subgroup of PUC <20 mm *N* = 15	Subgroup of PUC ≥20 mm *N* = 10
Age at 1st stage repair	Median = 12 months Range = 9 months–12 years		
Time interval between 1st and 2nd stage repair	Median = 9 months		
Meatus location			
Penoscrotal	12/34	7/15	3/10
Scrotal	10/34	5/15	1/10
Perineal	12/34	3/15	6/10
		PUC <20 mm vs. PUC ≥20 mm in meatus location *p*-value = 0.11	
Ventral curvature	Mean = 71 degrees SD = 16 degrees	Mean = 70 degrees SD = 18 degrees	Mean = 66 degrees SD = 12 degrees
	Median = 70 degrees Range = 45–90 degrees	PUC <20 mm vs. PUC ≥20 mm in ventral curvature *p*-value = 0.47	
DSD	4/34	0/15	3/10
45,X/46,XY	2		2
46,XY PGD	2		1
PUC detected by cystoscopy	25/34		
PUC longitudinal dimensions	Median = 15 mm Range = 10–35 mm		
Abnormal verumontanum	2/34	0/15	2/10
			Hypoplastic = 1
			Absent = 1
Symptomatic PUC	3/25	0/15	3/10
REO	1		1
Post-void dribbling	1		1
Pyuria	1		1
		PUC <20 mm vs. PUC ≥20 mm in symptomatic PUC *p*-value = 0.024	

PUC, prostatic utricle cyst; REO, recurrent epididymoorchitis; DSD, differences of sex development; PGD, partial gonadal dysgenesis.

**Figure 3:**
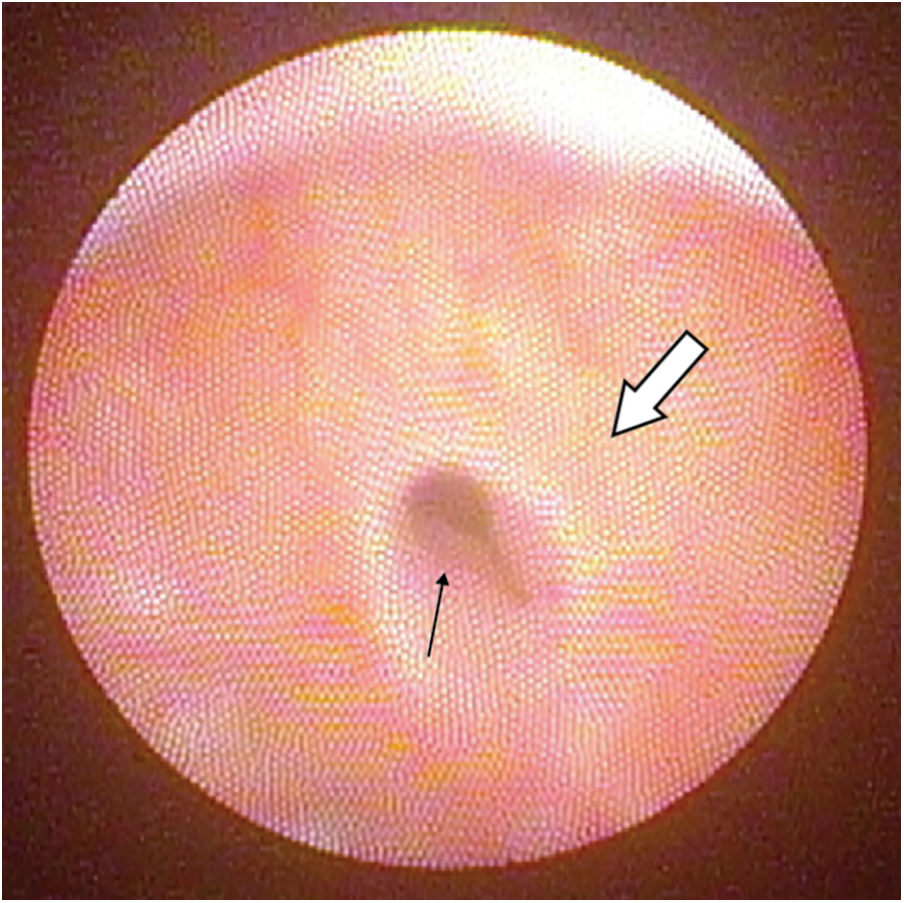
A small hypoplastic verumontanum(white arrow) with PUC opening(black arrow).

Clinical data of the 3 symptomatic patients are listed in Table 2. The first symptomatic patient presented with post-void urinary dribbling after completing toilet training. Dribbling varied in amount and usually happened within an hour after voiding. Parents reported that the urine being dribbled sometimes appeared turbid. The urinary stream was otherwise strong as demonstrated in the uroflowmetry without suspicion of obstruction. Cystoscopy was repeated with negative findings of urethral stricture, urethral diverticulum or meatal stenosis. The verumontanum was hypoplastic in this patient and the PUC opening allowed easy passage of the cystoscope (Figure 3). The patient was managed conservatively with the advice of massaging the perineum after voiding to facilitate expression of urine trapped in the PUC.

**Table 2:** Summary of the 3 patients with symptomatic PUC.

Age at 2nd stage of hypospadias repair	Patients 1	Patient 2	Patient 3
	23 months	22 months	20 months
Primary presentation of symptomatic PUC	Post-void dribbling after toilet training	Pyuria at 4 months after 2nd stage hypospadias repair	Recurrent epididymoorchitis since 10 months after 2nd stage hypospadias repair
PUC longitudinal dimension	35 mm	20 mm	30 mm
Verumontanum appearance	Hypoplastic	Normal	Normal
Co-morbidity	Nil	46,XY DSD due to PGD associated with *DMRT1* gene deletion	Nil
Treatment for PUC-related symptoms	Repeated cystoscopy to rule out urethral diverticulum or obstructive pathology associated with hypospadias surgery	Intravenous antibiotics	Intravenous antibiotics and Foley catheter insertion
			Cystoscopic widening of PUC opening and catheterization with aspiration
			Robot-assisted laparoscopic excision of PUC at age of 4 years
	Conservative management with advice on perineum massage after voiding		
At the latest follow-up	Observation for the persistent post-void dribbling	No recurrent episodes	Symptom-free >2 years
		Symptom-free >1 year	
	Practicing perineum massage after voiding		

PUC, prostatic utricle cyst; DSD, differences of sex development; PGD, partial gonadal dysgenesis.

The second symptomatic case was a 46,XY DSD patient with partial gonadal dysgenesis. The patient presented with one episode of atypical UTI with pyuria at 4 months after second-stage hypospadias repair, and required in-patient treatment with antibiotics. Repeated ultrasound examinations were unable to demonstrate the PUC detected by cystoscopy. Micturating cystourethrogram (MCUG) showed absence of vesicoureteral reflux or urethral stricture while pooling of contrast in the PUC was demonstrated in the voiding urethrogram. The patient did not have recurrence of UTI and was managed conservatively with regular follow-up.

The third symptomatic patient presented with recurrent episodes of epididymoorchitis since 10 months after second-stage hypospadias repair. The first few episodes were still manageable by antibiotic treatment. Subsequent episodes were associated with suprapubic and groin pain, and required bladder decompression by indwelling catheter in addition to intravenous antibiotics. We proceeded to intervene by performing cystoscopy and transurethral incision to widen the PUC orifice. We also cannulated the PUC for aspiration and culture. However, the patient still had recurrent symptoms after endoscopic treatment. Definitive surgery was offered and the parents were counselled about the possibility of disconnecting the vas deferens with definitive surgery. Robot-assisted laparoscopic excision of PUC was performed 3 years after hypospadias surgery. The bilateral vas deferens were noted to be connected with the PUC at its dome, and were thus disconnected during surgery. The patient remained free from symptoms for more than 2 years after definitive surgery and postoperative imaging did not reveal any residual PUC.

## Discussion

We found 74% of our patients presenting with proximal hypospadias had concomitant PUC diagnosed by cystoscopy. Our finding of the prevalence of PUC associated with hypospadias is higher than previous studies which reported a wide range of 9%–67% (2, 3, 9–13). We believe the high prevalence noted in the present study was attributable to both the severity of hypospadias of the study subjects, and the diagnostic tool we employed. An earlier study screened 280 hypospadias patients for PUC by retrograde urethrogram and/or micturating cystourethrogram. The authors reported that PUC was demonstrated in 27.5% patients (11). Using a combined approach of retrograde urethrogram plus ultrasonography, Kojima et al. found that PUC was present in 45% of their hypospadias patients (12). Both studies included patients with distal and proximal hypospadias. In another study the authors used urethrogram and reported PUC in 59% of scrotal and perineal hypospadias (3). While radiological investigations are standard of care in diagnosis of urinary tract anomalies, PUC may be missed by urethrogram or ultrasonography due to incomplete filling of the cavity or small size (9, 10, 12). Notably one of our symptomatic patients had repeated ultrasound examinations which failed to detect the PUC of 20 mm noted on cystoscopy. Our finding provides further support that cystoscopy may be more reliable than radiological investigations to detect PUC.

Data are limited in the existing literature regarding the use of cystoscopy as the diagnostic tool for PUC. Gupta et al. and Devine et al. reported that 33% and 57% of their patients with proximal hypospadias respectively had PUC noted on cystoscopy (2, 9). The authors of both studies described their cystoscopies as routine for proximal hypospadias, although they did not elaborate further on their protocols of including cystoscopy in proximal hypospadias management, nor did they specify the size of cystoscope (2, 9) Our idea of PUC screening by cystoscopy during hypospadias repair followed our experience of encountering patients with prior history of proximal hypospadias repair returning to us for symptomatic PUC. We selected proximal hypospadias patients who needed 2-stage repair as the target group to introduce PUC screening as we believed the diagnostic yield among these patients would be higher than patients with other forms of less severe hypospadias. Moreover, proximal hypospadias patients have higher risk of on-going problems, and thus the benefits of additional prognostic information of PUC should outweigh the risk of an extra procedure of diagnostic cystoscopy to be performed concurrently with hypospadias repair.

We advocate the use of the smallest cystoscope (Fr 4.5/6.5 or Fr 7.5) as the standard instrument for PUC screening. In contrast, others have reported the diagnostic criterion of having a Fr 9 or above cystoscope to be able to pass through the PUC opening at verumontanum in order to make the diagnosis (13). We believe an important implication in hypospadias patients would be an accurate detection of concomitant PUC and its dimension rather than the size of its orifice. A tiny or slit-like orifice is not reassuring if it is associated with a sizable PUC as such anatomy may result in progressive stasis and distension (12). Endoscopic widening of a small PUC orifice to improve drainage has been reported to be a successful treatment in select cases of symptomatic PUC (15). Our findings suggest that using the smallest-sized cystoscope can enhance the diagnostic yield by detecting PUC with small openings which would otherwise have been missed had a bigger scope been used.

Howard was the first to report a direct relationship between the severity of hypospadias and the increasing size of PUC (16). His finding was supported by subsequent studies which demonstrated a higher prevalence of PUC in proximal hypospadias and an association of large PUC with proximal hypospadias (2, 11). We did not find any association of the size of PUC with the meatal location or the degree of ventral curvature among our study subjects. However, our analysis was limited by the small number of only 10 cases of PUC ≥20 mm, and the fact that all the study subjects had the severe form of hypospadias made subgroup analysis unlikely to reach statistical difference.

Ikoma et al. classified PUC based on the urethrogram findings almost 4 decades ago (11), and the classification is still being used to this day (4, 9). According to their classification, the openings of PUC of Grade 0, I, and II are located in the posterior urethra. PUC is classified as grade 0 if it is confined within the verumontanum and Grade I if it extends above the verumontanum but still below the bladder neck. PUC of Grade II has its proximal extent above the bladder neck. PUC is classified as Grade III if its opening is located at the bulbar urethra regardless of its proximal extent (11). We did not cannulate PUC to perform contrast study for grading during cystoscopy as described by other investigators (9). We postulate that the endoscopic features may prove to be similarly useful for assessing the risks of symptom or complication development after hypospadias repair. Oh et al. examined 50 cadaveric specimens of the adult prostate gland. The authors reported that the length of normal variants of the prostatic utricle ranged from 6 to 12 mm, and width 0.8–3.5 mm (1). The 25 PUC reported in our study had longitudinal dimensions of 10–35 mm at the age of 1–2 years. Given the young age of our patients, we believe that even PUC of 10 mm in longitudinal dimension should be considered as an anomaly instead of a normal variant. It is of note that cystoscopy was able to demonstrate a cystic cavity in our 25 patients with positive findings in contrast to a narrow structure described in normal variants (1).

Three out of 25 patients (12%) developed symptoms possibly related to PUC in our study. Our finding is in agreement with another study that reported symptom development in 14% of PUC after proximal hypospadias surgery (9). A long-term longitudinal study would likely find a higher incidence as symptomatic PUC have been reported in young adults or adolescents years after their initial hypospadias surgery in early childhood (17–19). Late onset of symptoms can also occur in PUC of small size (17). Although we did not note a higher incidence of symptomatic PUC than previously reported, our finding of increased risk of symptom development in PUC ≥20 mm suggests that proximal hypospadias children having PUC ≥20 mm detected by cystoscopic screening need special attention. Most of the symptomatic PUC cases reported in literature had prior history of proximal hypospadias repair (5, 6, 9). Proximal hypospadias repair creates a long neourethra which significantly increases the distance of urine passage. The neourethra may not be as compliant as the native urethra, and varying degrees of functional obstruction inherent with a long neourethra may predispose to stasis in PUC even in the absence of mechanical obstruction such as urethral stricture. It is logical to postulate that the bigger the size of PUC, the higher would be the risk of symptom development when progressive stasis occurs.

Our 3 symptomatic patients had PUC dimensions of 20, 30 and 35 mm respectively. Acimi et al. reported in their recent study that 10 out of 11 cases of symptomatic PUC associated with severe hypospadias had a dimension of 20 mm or more measured by urethrogram (3). The size of a PUC shown in urethrogram may vary with the degree of its distension by contrast filling. We believe that cystoscopy may be a better tool to achieve consistent measurement of the dimension of PUC. We are cautious about making any recommendations on the follow-up management of PUC detected by cystoscopic screening as evidence on preventive measures or interventions are still lacking. However, we believe it would be prudent to consider close follow-up particularly for those who have PUC ≥20 mm in the first few years after hypospadias surgery, and to take proactive approach to exclude or to manage early any obstructive complications of urethroplasty before progressive stasis occurs in PUC and symptoms develop. Parents should be well informed of the positive findings of PUC and be cautioned about its potential complications.

We noted an abnormal verumontanum in 2 patients out of the whole group. One was a symptomatic patient with flat hypoplastic verumontanum, and the other patient had sex chromosomal DSD with 45,X/46,XY karyotype. The latter had no verumontanum noted on cystoscopy and the PUC opening was located at the bulbar urethra which corresponded to Grade III in Ikoma's classification. The finding of a PUC opening at the bulbar urethra has raised our concern as urine trapped in his PUC may not be controlled by the continent mechanism of the external sphincter. The patient has yet to complete toilet training and we are uncertain if he will present with symptoms of post-void dribbling. In a recent study, cystoscopy was performed in 12 patients who had proximal hypospadias and had PUC identified by urethrogram (4). The authors reported hypoplastic verumontanum in 4 cases and absent in 1 case. The authors did not identify any correlation between the karyotype with the anomalous verumontanum (4). Clinical relevance of an abnormal verumontanum in the context of PUC remains yet to be answered.

One of our symptomatic patients had an atypical presentation of UTI with pyuria. Stasis of infected urine within a confined cavity of PUC can present with purulent discharge or pyuria which has been reported before (5–7). This patient did not experience recurrence of symptoms and was managed conservatively. MCUG findings had also excluded urethral stricture or vesicoureteral reflux in this patient. Urethral obstruction after hypospadias surgery needs to be excluded in symptomatic PUC. Surgical correction of urethral stricture alone has been reported to be successful in resolving PUC symptoms in select cases (9).

Recurrent epididymoorchitis is one of the common indications for surgical intervention for PUC (3, 6, 9, 20). Initially, we managed our patient with recurrent epididymoorchitis conservatively. Given his recurrent symptoms with increasing severity, we decided to perform a transurethral incision to widen the PUC opening and catheterize and aspirate the PUC using a similar technique as previously described (6, 15, 20). However, his symptoms recurred soon after endoscopic treatment. He remained symptom-free only after definitive surgery involving PUC excision by a robot-assisted approach. We also encountered in this case the challenging anatomical variation of having both vas deferens connected to the dome of the PUC, and as a result both were transected in the same way as previously reported (6, 7, 9).

We recognize the limitations of our study which was a retrospective review of the medical records and video recordings of cystoscopy were not available in all study subjects for review. There was a lack of description of the details of verumontanum and PUC orifice in our data. Both interobserver and intraobserver variability might exist in the interpretation of the cystoscopy findings although all of the procedures were performed by the two authors.

## Conclusion

Our study suggests that PUC is highly prevalent in children with proximal hypospadias, and using the smallest-sized cystoscope as a screening tool can increase the diagnostic yield. Although the incidence of symptomatic PUC among our patients is low, the relatively short follow-up of the present study has precluded conclusions of the long-term risks which are expectedly higher given the reports of late onset of symptoms. Integrating cystoscopy in hypospadias surgery for concurrent PUC screening can be considered as an option for patients with proximal hypospadias. PUC ≥20 mm may be associated with an increased risk of subsequent symptom development which should be emphasized in parental counselling. PUC screening in proximal hypospadias children is a debatable subject. Prospective multi-institution studies would definitely help to provide more robust data to this subject which is highly relevant but consensus is still lacking.

## Data Availability

The datasets presented in this article are not readily available because access to the database is not allowed outside our institution. Requests to access the datasets should be directed to YH Tam, pyhtam@surgery.cuhk.edu.hk.
